# Mapping of the central sulcus using non-invasive ultra-high-density brain recordings

**DOI:** 10.1038/s41598-024-57167-y

**Published:** 2024-03-19

**Authors:** Leonhard Schreiner, Michael Jordan, Sebastian Sieghartsleitner, Christoph Kapeller, Harald Pretl, Kyousuke Kamada, Priscella Asman, Nuri F. Ince, Kai J. Miller, Christoph Guger

**Affiliations:** 1 g.Tec Medical Engineering GmbH, Schiedlberg, Austria; 2 Institute for Integrated Circuits, Johannes Kepler University, Linz, Austria; 3 Institute of Computational Perception, Johannes Kepler University, Linz, Austria; 4 Department of Neurosurgery, Megumino Hospital, Eniwa, Japan; 5 Department of Biomedical Engineering, University of Houston, Houston, TX USA; 6 Department of Neurosurgery, Mayo Clinic, Rochester, USA

**Keywords:** Neuroscience, Motor control, Sensorimotor processing, Somatosensory system, Biomedical engineering

## Abstract

Brain mapping is vital in understanding the brain’s functional organization. Electroencephalography (EEG) is one of the most widely used brain mapping approaches, primarily because it is non-invasive, inexpensive, straightforward, and effective. Increasing the electrode density in EEG systems provides more neural information and can thereby enable more detailed and nuanced mapping procedures. Here, we show that the central sulcus can be clearly delineated using a novel ultra-high-density EEG system (uHD EEG) and somatosensory evoked potentials (SSEPs). This uHD EEG records from 256 channels with an inter-electrode distance of 8.6 mm and an electrode diameter of 5.9 mm. Reconstructed head models were generated from T1-weighted MRI scans, and electrode positions were co-registered to these models to create topographical plots of brain activity. EEG data were first analyzed with peak detection methods and then classified using unsupervised spectral clustering. Our topography plots of the spatial distribution from the SSEPs clearly delineate a division between channels above the somatosensory and motor cortex, thereby localizing the central sulcus. Individual EEG channels could be correctly classified as anterior or posterior to the central sulcus with 95.2% accuracy, which is comparable to accuracies from invasive intracranial recordings. Our findings demonstrate that uHD EEG can resolve the electrophysiological signatures of functional representation in the brain at a level previously only seen from surgically implanted electrodes. This novel approach could benefit numerous applications, including research, neurosurgical mapping, clinical monitoring, detection of conscious function, brain–computer interfacing (BCI), rehabilitation, and mental health.

## Introduction

Mapping the human cortex is crucial for understanding brain function and identifying regions associated with behavior and disease. Multiple technologies are currently available to perform cortical mapping, including electrocorticography (ECoG), electroencephalography (EEG), functional magnetic resonance imaging (fMRI), electromagnetic imaging (MEG), near-infrared spectroscopy (NIRS)^1^, and stereoelectroencephalography (SEEG)^2^. ECoG, SEEG, and EEG provide a high temporal resolution, a significant advantage in diagnostic tools for measuring brain activity in clinical neurophysiology^3,4^. However, invasive cortical recordings using ECoG or SEEG approaches are only applicable after surgical implantation, and their usage is inevitably connected to risks for the patient and increased costs in general^5,6^. In contrast, EEG technologies are inexpensive and straightforward to implement in a simple laboratory setup. Therefore, researchers and clinicians are interested in overcoming limiting factors of EEG, such as the low spatial resolution and susceptibility to artifacts that often originate from movement and electromyographic (EMG) activity^7^.

The EEG signal measured from each electrode on the scalp’s surface EEG reflects summed up postsynaptic potentials. Additionally, these potentials are filtered by tissue, bone, and vessels when passing through the scalp^8^. This accumulation of impedances from different conducting and insulating tissue planes acts as an anisotropic spatial lowpass filter^9^. Analogous to the classical Nyquist rate in temporal bandwidth estimation when determining the optimal number of sample points, ‘spatial Nyquist rates’ may allow an estimate of the optimal number of spatially distributed sensor positions^10^. Initial work in finding the optimal EEG electrode positioning suggested that an electrode distance of 20–30 mm (128 sensors distributed over the whole scalp) is sufficient to extract the maximum possible information from EEG signals and achieve the spatial Nyquist rate^9,10^. Robinson et al.^11^ found that more fine-grained patterns of neural activity (i.e., patterns with higher spatial frequencies) can be detected over the occipital areas with an interelectrode distance of 14 mm compared to a standard setup with approximately 28 mm electrode distance. In response, so-called multichannel or high-density EEG (HDEEG) systems have been constructed with more sensors distributed at a higher density over the area of interest or the whole scalp, offering greater spatial coverage and higher temporal resolution^6^. HDEEG has subsequently proven helpful for patients with epilepsy during preoperative localization of epileptogenic lesions using source localization^12–14^, identifying epileptic high-frequency oscillations (HFO) and mapping language cortex^15^. HDEEG has found use in other clinical settings, such as improved diagnosis and quantification of cognitive disorders after acute brain injury^16^ and spatially locating brain activation during dreaming in non-rapid eye movement in sleep^17^. Moreover, high-density EEG can improve recognition accuracy when characterizing human emotional states using brain-directed connectivity (BDC) networks compared to standard (low-density) EEG^18^.

However, recent studies have found that these HDEEG systems do not have enough electrodes to thoroughly record and interpret EEG^11,19,20^. Grover and Venkatesh analyzed the spatial bandwidth of EEG to determine how many sensors need to be distributed spatially using an information-theoretic approach. They proposed that dramatically increasing the sensor points from a few hundred to a few thousand transforms EEG to a quantity different level of sensing and source-localization ability^20^. We tested this hypothesis with human EEG data.

In this manuscript, we describe a novel ultra-high-density EEG (uHD EEG) system created using flexible printed circuit boards (PCBs) with gold-plated electrode points (Fig. 3). With an inter-electrode distance of 8.6 mm and an electrode diameter of 5.9 mm, the density of the electrodes exceeds all commercially available EEG systems. We use this system to demonstrate electrophysiological localization of the central sulcus on the brain beneath, revealing a functional precision in brain mapping from the scalp that has previously only been possible using surgically implanted electrodes that directly contact the brain. The uHD EEG under investigation has been previously employed in two antecedent studies, demonstrating its prior utilization for single finger movement decoding^21^ and hand gesture decoding purposes^22^.

Intraoperatively neurophysiology (ION) techniques are the gold standard for monitoring brain function and spatial mapping of brain areas. During surgery, procedures for generating somatosensory evoked potentials (SSEPs) are often carried out as they provide essential spatial information about the somatosensory cortex and the central sulcus (CS)^3,23,24^. Mainly, they are used to prevent any postoperative neurological deficits due to the resection of functional anatomical regions^25^. Furthermore, SSEPs can help with the prognosis of recovery success after traumatic brain injury^26^. In addition, SSEPs can provide beneficial information indicating hand and bladder function and recovering ambulation in spinal cord injury^27^. Generally, evoked potentials are considered the nervous system’s response to sensory stimulation, such as auditory, visual, and somatosensory stimuli. SSEPs, as a result, reflect an accumulation of a series of waves that show activation of neural structures along the somatosensory pathways. SSEPs can be elicited from mechanical stimuli as well as from electrical stimulation. However, to receive more prominent and robust neural responses, electrical stimulation of peripheral nerves is favored in clinical studies^28^. The somatosensory evoked potential phase reversal (SSEP-PR) phenomenon is characterized by the cortical N20 potential when detected from the postcentral gyrus, and the cortical P20 potential from the pre-central gyrus is a widely applied methodology to identify the somatosensory cortex^29,30^.

Induced by the median nerve stimulation (MNS), the primary cortical SSEP component in the somatosensory cortex is an N20 near-field potential located over the parietal area contralateral to the stimulated nerve. This potential mainly reflects neural activity in the primary somatosensory cortex (S1), which is responsible for receiving peripheral sensory information. Still, it relies on the secondary somatosensory cortex (S2) to process and store this information. The S2 region is connected to the hippocampus and amygdala, enabling it to receive environmental input and make decisions by integrating prior experience and current appraisal of the stimulus. The output is then directed to other brain areas, assisting higher-order processing and problem-solving^31^.

A phase reversal of this potential is visible, moving from the sensory area S1 (pre-central gyrus) toward the post-central gyrus to the area associated with motor planning tasks (motor cortex M1). See Fig. 4b for an overview of the phase reversal principle with the functional brain area and corresponding evoked potential marked in blue (somatosensory (S1) cortex), red (motor (M1) cortex), and the central sulcus marked in green. If the electrode is positioned pre-central to the CS, the neural response is expected to appear first as a positive peak at around 20 ms (P20) post-stimulus, followed by a significant peak around 30ms after the electrical stimulation (N30). If the electrode is over the postcentral gyrus, the response will be identifiable by a negative peak around 20 ms (N20) followed by a positive peak around 30 ms after the stimulus (P30). Located over the central sulcus, the neural response acquired by the electrode generally forms a delayed wave forming the 1st positive peak at 25 ms (P25) with a lower amplitude, followed by an N30–35 negative deflection of the EP^3,32,33^.

Simultaneous recording of the neural activity from the ipsilateral cortical surface allows cortical sensory mapping (CSM) and cortical motor mapping (CmM)^29^. The peripheral stimulation of the median nerve is commonly done by applying bipolar surface electrodes in the area of the distal wrist^34^. The SSEP-PR from anterior to posterior to the CS was adopted in this study to locate the primary motor and the somatosensory cortex. This paper shows the application of a novel uHD EEG system used to record SSEPs for the mapping of the CS.

## Results

**Figure 1:**
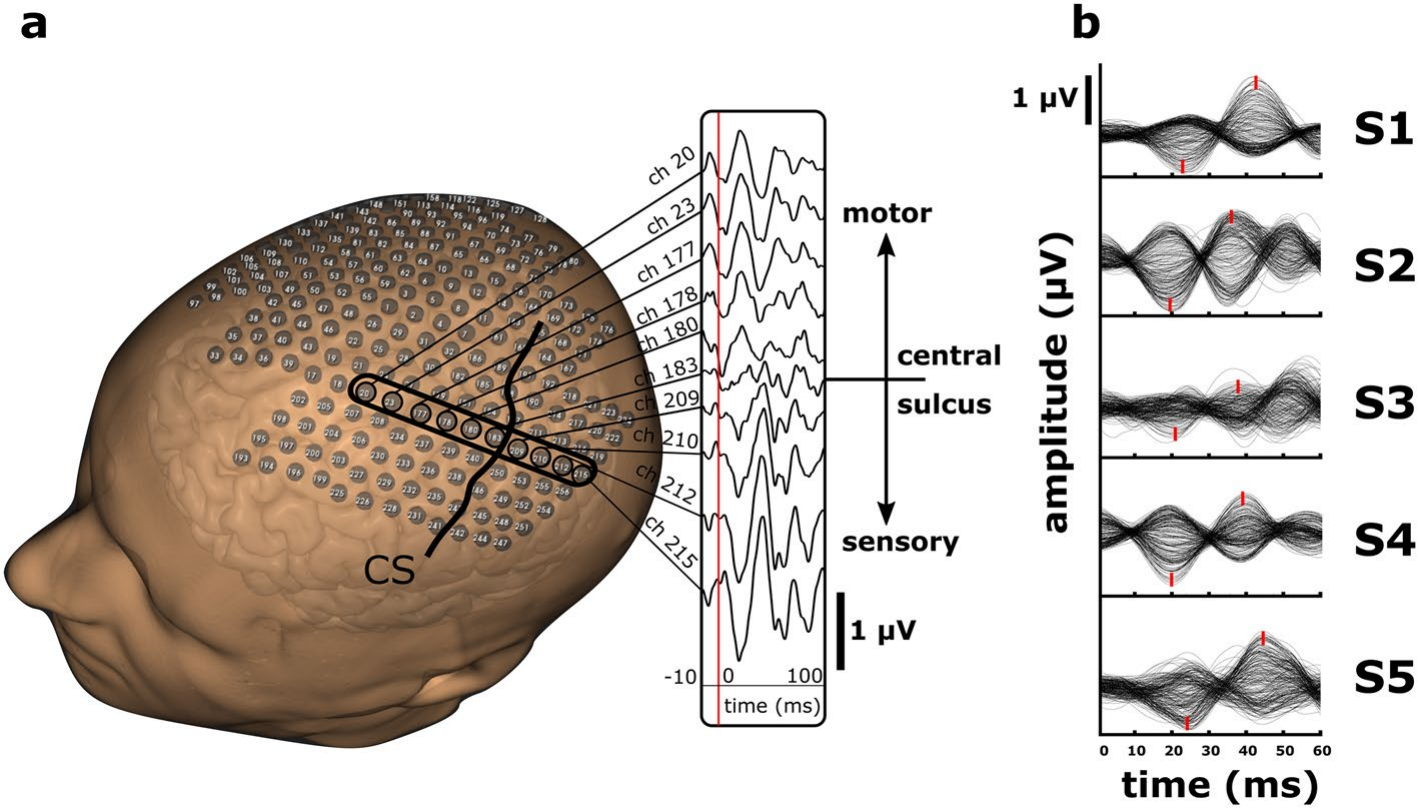
SSEPs evoked by MNS. (**a**) Spatial distribution of SSEPs across a row of 10 channels. The 3D head model displays the total number of 256 channels from the uHD EEG electrode montage. Bold encircled channels were selected for visualization of the phase reversal. Channels anterior to the Central sulcus (CS)—ch. 20, 23, 177, 178, 180, show positive peaks followed by a negative waveform. Channel 183 exhibits reduced deflection and delayed response, indicating its location on the central sulcus. In contrast, channels posterior to the central sulcus—ch. 209, 210, 212, and 215 show the first negative deflection followed by a positive waveform. (**b**) Raw SSEP traces from each subject show the response variability across individuals with peak timing marked in bold red.

Scalp SSEPs show phase reversals in close proximity to the central sulcus. As depicted in Fig. 1a, the phase reversal is marked across ten sensor locations placed anterior, posterior, and on the CS. Raw SSEPs from a right-hand MNS acquired from sensor locations identified on the left hemisphere are drawn for S1. The red line represents the stimulation onset with a time axis from − 10 to 100 ms post-MNS, and the y-axis depicts µV with limits of ± 1 µV. Channel numbers 20, 23, 177, 178, and 180 show P22 and N40 (1st positive peak with 22 ms latency, 1st negative peak with 40 ms latency). Channel 183 represents a channel’s response that is close to the CS with a decrease in signal amplitude and higher latencies, resulting in a P27 and N40 response. Channel numbers 209, 210, 212, and 215 are expected to be posterior to the CS with similar but reversed timing of the SSEP, resulting in a 1st negative N22 and 1st positive P41.

Fig. 1b displays SSEP traces from all five subjects. The plots’ amplitude range is set to ± 1 µV, and the time ranges from stimulus onset until 60 ms post-MNS. The timing of positive and negative SSEP peaks varies across all subjects. Physiological peak latencies for the 1st N and 2nd P show slight differences across subjects. Latencies from the first N peak were on mean (SD) 21.1 (1.6) ms post-stimulus. The signal peak of the second P peak has an average latency of 39.6 (2.4) ms post-stimulus.

SSEPs over the frontal lobe can be separated from those over the parietal lobe. Classification results acquired from applying both the spectral clustering and peak detection methods are listed in Table 1. The table shows the accuracies obtained from channels classified either anterior or posterior to the central sulcus. On average, an accuracy of 95.2% was achieved in classifying anterior vs. posterior channels using the spectral clustering method. Accuracies range from 90.6 to 97.2% for subjects S5 and S4, respectively. The peak detection method resulted in an average accuracy of 91.9 %, with 86.6% being the lowest and 96.6% being the highest for subjects S3 and S1, respectively.

**Table 1:** Classification results from anterior and posterior classified channels using spectral clustering and peak detection.

Classification results	S1		S2		S3		S4		S5		Average	
	Ant.	Pst.	Ant.	Pst.	Ant.	Pst.	Ant.	Pst.	Ant.	Pst.	Ant.	Pst.
n (truth; Ant. or Pst.)	153	98	124	114	137	116	145	105	100	155	131.8	117.6
n (classified; Ant. or Pst.) spectral clustering	160	91	126	112	131	122	146	104	108	147	134.2	115.2
Accuracy spectral clustering	96.4%		96.6%		95.2%		97.2%		90.6%		95.2%	
n (classified; Ant. or Pst.) peak detection	149	102	106	132	144	109	134	116	127	128	132	117.4
Accuracy peak detection	96.6%		92.6%		86.6%		94.3%		89.5%		91.9%	

The table compares the channels as anterior or posterior to the central sulcus using the spectral clustering and peak detection method for five subjects. The number of ground truth channels for each subject is shown in row 1. The number of channels classified as anterior or posterior for each subject using spectral clustering and peak detection are given in rows 2 and 4, respectively. Rows 3 and 5 show the final classification accuracy with the average accuracy across all subjects in the last column.

Table 2 reports SSEP latencies for channels categorized as Ant. and Pst. using the peak detection method. The table presents the mean and standard deviation in milliseconds for each subject, covering anterior classified channels for the first positive (1st P) and second negative (2nd N) peaks, as well as posterior classified channels for the first negative (1st N) and the second positive (2nd P) peak.

**Table 2:** SSEP latencies from anterior and posterior classified electrodes for all subjects.

S1				S2				S3				S4				S5			
Ant.		Pst.		Ant.		Pst.		Ant.		Pst.		Ant.		Pst.		Ant.		Pst.	
1st P	2nd N	1st N	2nd P	1st P	2nd N	1st N	2nd P	1st P	2nd N	1st N	2nd P	1st P	2nd N	1st N	2nd P	1st P	2nd N	1st N	2nd P
22.4 (3.6)	40.6 (5.1)	21.6 (4.0)	41.1 (3.2)	20.4 (2.8)	34.8 (6.9)	19.4 (6.9)	36.3 (2.8)	20.9 (3.4)	41.2 (5.3)	20.6 (3.6)	39.4 (4.0)	17.9 (2.7)	35.8 (3.2)	22.5 (2.6)	38.6 (4.3)	20.3 (1.6)	45.9 (1.42)	22.1 (1.3)	42.6 (3.4)

The table presents results from the analysis of latency statistics for the first and second negative and positive SSEP peaks across anterior (Ant.) and posterior (Pst.) electrodes, classified using the peak detection approach. Latency values are expressed in milliseconds as mean (SD).

**Figure 2:**
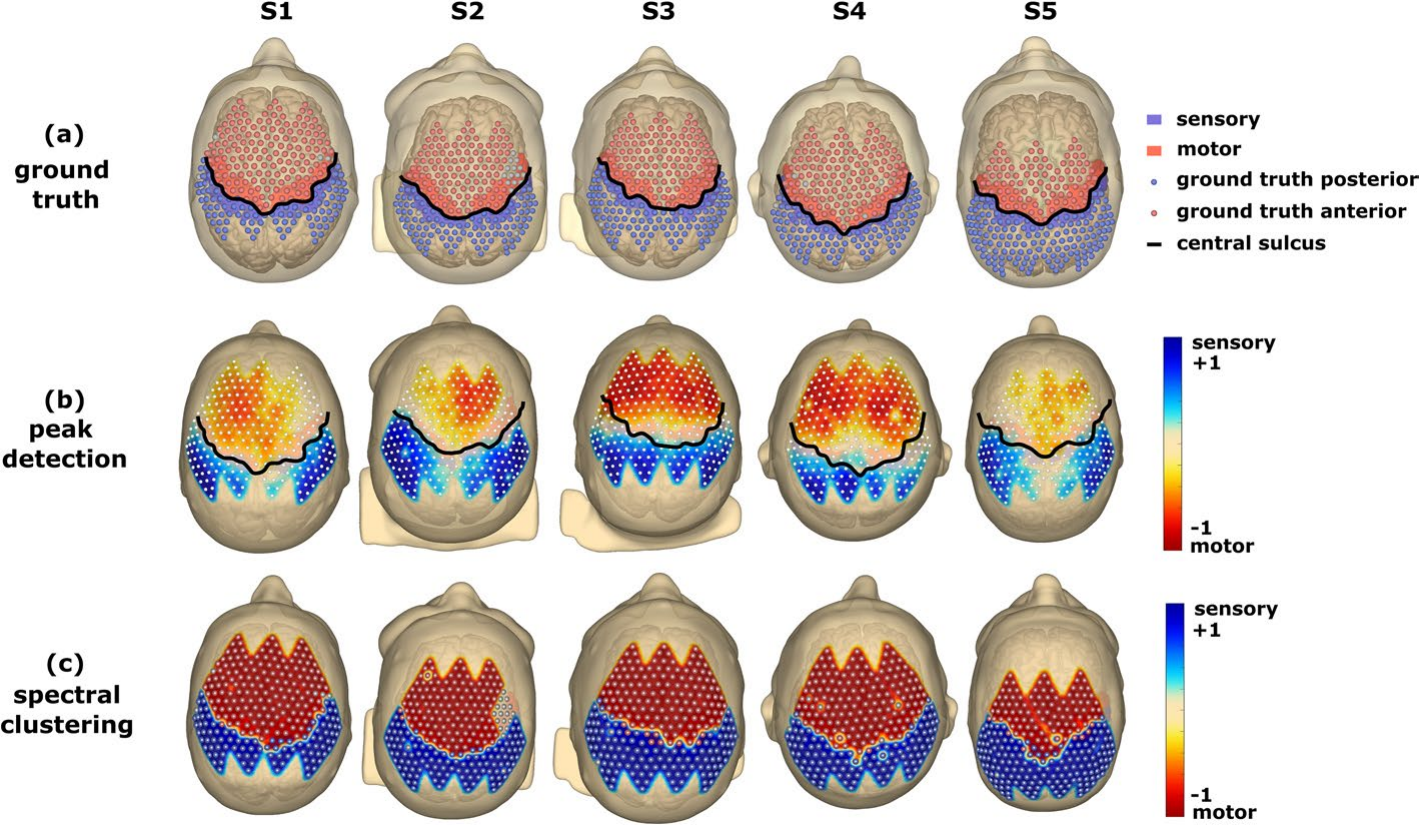
Representation of ground truth and heatmaps generated from SSEP classification. (**a**) The first row of head models represents the ground truth of channel distribution for S1 to S5, respectively. The anatomically correct motor area M1 is marked in red on the cortex of the MRI, while the sensory cortex is colored blue. The central sulcus, which delineates the sensory and motor cortex, is highlighted in bold black. The number of 256 channels on the head model is indicated by small spheres, with red spheres representing channels expected to be located anterior to the central sulcus and blue spheres representing those posterior to the central sulcus. Grey spheres represent channels with bad signal quality. (**b**) The second row shows the results of the peak detection method, with the results interpolated across the scalp using normalized values from 1 (blue)—maximum sensory peak to − 1 (red)—minimum motor peak. The central sulcus is marked similarly to the ground truth, with bad channels excluded from interpolation. (**c**) The results from classification using spectral clustering for subjects S1 to S5 can be seen in the third row. As the clustering is a 2-class problem, there was only a decision between either 1 (blue)—sensory or − 1 (red)—motor. Therefore, bad channels are depicted in grey.

The final results of the channel classification using peak detection and spectral clustering are shown in Fig. 2. Figure 2a depicts the head models and the channel distribution of the ground truth for subjects S1–S5. Anatomically correct motor and sensory areas (M1 and S1) are denoted by red and blue coloring on the cortex of the MRI, with the central sulcus highlighted in bold black to separate them. The 256 total channels on the model are represented by small spheres, with red and blue spheres indicating anterior and posterior channel locations, respectively, and grey spheres indicating channels with poor signal quality. Figure 2b shows results from the peak detection method, which are interpolated across the scalp and colored on a scale from blue (maximum sensory peak) to red (minimum motor peak), with bad channels excluded. Values range from − 1 to 1, as explained in the methods section. The third row (Fig. 2c) shows results from spectral clustering classification, with a decision between sensory (blue) or motor (red) for each channel. The classification problem was binary and, therefore, solely resulted in 1 and − 1 decisions for predictions of each respective channel.

## Discussion

### Ultra-high-density EEG system

The majority of current systems employed for high-density surface-EEG measurements in humans rely on caps or similar stretchable structures to position the electrodes. These attachment mechanisms and caps impose spatial limitations, restricting the achievable electrode density. Furthermore, wet electrode technologies encounter obstacles in achieving higher densities due to the presence of bridges between electrodes, leading to undesirable crosstalk among channels. Additionally, challenges arise from the lack of well-defined contact areas resulting from the inconsistent distribution of conductive gel or saline electrolyte solutions, impeding reproducibility in EEG experiments and source localization. Consequently, the critical aspects for successful high-density EEG investigations lie in the proper differentiation of channels and the consistent maintenance of low impedance levels throughout the study^35^.

The proficient utilization of the uHD EEG system has demonstrated compelling evidence that adopting novel methodologies effectively addresses these constraints, leading to notable enhancements in electrode density and signal acquisition reliability.

The results presented in this study support findings in prior research indicating that increased electrode density can aid in the interpretation of brain signals. For instance, Robinson et al. observed improvements in evaluating the spatiotemporal aspects of early visual processing by enhancing spatial density, referred to as super Nyquist density (SND), with a sensor distance of 14 mm^11^. Similarly, Scarff et al. showed that enhancing the recording setup from 64 to 128 electrodes resulted in improved accuracy for auditory evoked potentials (AEPs) using equivalent dipole source localization^36^. We believe that the robustness of evoked potentials makes them particularly suitable for improving analyses with higher electrode densities. However, as stated in the introduction, the volume conduction limits the interpretation by just using the scalp EEG and anatomical information. Therefore, we would like to emphasize the significance of employing source reconstruction methods to enhance accuracy. Earlier work by Lantz et al. suggested that interictal epileptiform activity acquired by higher density is beneficial for reconstructing the sources and highlights the need for higher EEG density for high source location accuracy in epileptic patients^37^. Similar to Michel and Brunet^38^, we believe that the combination of high-density EEG systems, precise head anatomy information, and sophisticated source localization algorithms can evolve EEG into a genuine neuroimaging modality.

Highlighting the importance of effective use, users should be aware that the system performs optimally when hair is shaved, underscoring the necessity for this practice. Thorough testing of the system’s performance across diverse hair types and styles is crucial, with recognition of potential limitations. Notably, the system’s effectiveness decreases as hair length increases**.** Furthermore, it is important to note that as the number of channels (electrode grids) increases, challenges arise in the form factor of the uHD EEG system, which can lead to a prolonged setup time for sensor placement and a decline in user comfort.

### Spatial SSEPs

In Fig. 1a, the spatial distribution of SSEP traces for subject S1 can be seen on the head model reconstructed from the MRI scan. The sensor locations are placed anteriorly and posteriorly on the CS. Notably, a phase reversal is observed across ten sensor locations. This suggests a change in the polarity of the SSEP waveform at these specific locations. The phase reversal phenomenon could be attributed to the differential activation of neural populations within the somatosensory cortex, as found by several studies^26,30,39,40^. Compared to ECoG SSEP studies, the traces acquired from EEG show reduced amplitude but similar latencies, as found in similar studies^41^. Channels 209, 210, 212, and 215, expected to be located posterior to the central sulcus, exhibit the desired SSEP waveform with a negative peak (N) followed by a positive (P). This pattern aligns with the typical cortical response seen in somatosensory processing^3,30^. These findings indicate the successful activation of sensory pathways and the propagation of neural signals across the primary somatosensory cortex.

A detailed examination of peak latencies is presented in Table 2. Channels were categorized based on the peak detection method. For posterior (Pst.) selected channels, the average (SD) latency for the first N peak across subjects was 21.1 (1.6) ms post-stimulus, and the second P peak had an average latency of 39.6 (2.4) ms post-stimulus. Channels classified as anterior to the central sulcus (Ant.) exhibited an average (SD) latency of 20.4 (1.6) ms for their first P peak and 39.7 (4.5) ms for their second N peak. The second N peak of the anterior classified channels significantly varies across subjects with an SD of 4.5 ms. These inter-subject differences in peak latencies appear in comparable studies and may reflect variations in neural conduction velocities or discrepancies in the processing mechanisms of the somatosensory systems. However, the timing of the 2nd N peak has generally higher latencies, as found in comparative literature^3,26,30^. This could be explained by the lower bandpass cutoff at 20 Hz, which is lower compared to other studies but has shown the best results for delineation with the uHD EEG.

The SSEP response observed from channel 183 provides insights into the cortical activation near the central sulcus. Sensors above the CS show low peak information and the least activity from all selected channels. Latencies appear to be higher, with 27 and 40 ms for the 1st P and N peaks, respectively. This is consistent with findings in interoperative studies delineating the somatosensory motor cortex^3,42,43^.

Moving posteriorly to channels 209, 210, 212, and 215, which are expected to be located posterior to the central sulcus, the waveform reflects a P followed by the N peak, which aligns with the literature^3,24^. Similar to the posterior channels, slight SSEP timing and amplitude variations are observed. The timing is consistent with the reversed peak and shows the same latencies, which results in the typical butterfly plots as seen in Fig. 1b. The consistent amplitude range of ± 1 µV and the time portrayal from stimulus onset to 60 ms post-stimulation facilitate a comparative analysis of SSEP responses across subjects. The variations in timing and amplitude of the SSEP peaks among the subjects further emphasize the importance of considering individual differences in interpreting SSEP data.

The results presented in Fig. 1 provide valuable insights into the spatial distribution and characteristics of SSEP traces. SSEPs are widely used to assess the integrity and functionality of sensory pathways in the brain following sensory stimulation^3,25–27,30^. The observed phase reversal, differential responses across sensor locations, and inter-subject variations in peak timing and amplitude highlight the complexity and individual variability of the somatosensory system. Especially in non-invasive recordings, signal amplitude changes resulting from a low SNR can be problematic. Compared to ECoG data, we reached lower amplitudes but still prominent SSEP characteristics in the traces that could be used for further classification procedures.

### Delineation using peak-detection

The outcomes of the channel classification process using peak-detection and spectral clustering methods, as shown in Fig. 2, are visualized with the ground truth as the base for analysis. Therefore, the first row of Fig. 2 depicts head models, representing the ground truth of channel distribution for Subjects S1 to S5. Using the co-registration procedure and creating 3D head models allowed us to identify the right anatomical locations. The motor and sensory areas are correctly identified and visually distinguished on the MRI cortex in these models. The central sulcus, highlighted in bold black, is the boundary between the post-and pre-central gyrus.

Automatic approaches are vital, since manual inspection of the SSEP traces holds little utility in mapping procedures due to the high number of channels and expertise needed. The implementation of the peak detections represents a simple but computationally effective method for distinguishing SSEP morphologies. For better discrimination, the results are interpolated across the scalp and color-coded on a gradient scale ranging from blue (indicating the maximum sensory peak) to red (showing the maximum motor peak) (see Fig. 2, 2nd row). The normalized peak values allow visualization in a range of − 1 to + 1 and scalp color at 0, which allows simple visual detection of the areas with the highest activation. We have obtained satisfactory results in separating the pre- and postcentral using peak detection by drawing topographies on the scalp. The average accuracy, employing binary classification on peak-detection data, was 91.9% across all subjects. Nevertheless, caution is advised when interpreting accuracy, considering the expansive scalp area with low activation, particularly around the central sulcus, which is especially evident in subject S3, with a comparably low accuracy of 86.6%.

### Classification adopting spectral clustering

The third row of Fig. 2 showcases the results from the spectral clustering classification. Based on the clustering algorithm, each channel is assigned a decision, either sensory (blue) or motor (red). This 2-class approach allows simple but effective delineation of the central sulcus. Unsupervised spectral clustering was utilized to differentiate between the anterior and posterior channels. Spectral clustering employs subspace decomposition on high-dimensional data to cluster the information. Previous studies have used spectral clustering to delineate the central sulcus using ECoG and SSEPs^30^. It has also been employed to analyze channel grouping during different seizure stages by assessing their average mutual interactions^44^.

Additionally, spectral clustering has been used to assist in determining the epileptic focus by extracting features that cluster brain regions based on their functional dependencies^45^. The cortical areas used in this study included both pre- and post-centrally located channels for all of the subjects, which provided practical input for the machine learning framework. As mentioned by Asman et al.^30^, the presence of a single cluster confirmed improper CS crossing or the absence of a clear phase reversal. Therefore, applying the method with a small grid or strip ECoG electrodes can serve as an additional precaution when the grid has not crossed the CS or is poorly placed. The viability of this method (which was initially developed for intracranial recordings) for our uHD data opens a wide range of possible applications for the novel uHD EEG system.

On average, an accuracy of 95.2% was achieved in correctly classifying the anterior and posterior channels, which indicates comparable results with the same methodology applied on large high-density ECoG grids^30^. Additionally, the accuracy was calculated from 256 channels (excluding bad channels), which is more sensors than large high-density grids and therefore supports the accuracy analysis’s outcome. Adding up the number of incorrectly classified channels across the total number in mean (SD) resulted in 7.2 (4.7) for anterior incorrectly classified and 4.8 (3.1) for posterior incorrectly classified channels. Interestingly, subject S5 was an outlier with 16 and 8 incorrectly classified channels for anterior and posterior, respectively.

The results demonstrate that the spectral clustering method can accurately differentiate between the two regions based on the given data set. The high accuracies obtained indicate the robustness and reliability of the classification approach. The ability to successfully classify the channels as anterior or posterior has important implications for understanding the brain’s functional organization. It can aid various applications, such as brain-computer interfaces (BCI) or neuroimaging studies^46^.

Overall, the classification results highlight the potential of combining two methods—spectral clustering and uHD EEG—to comprehensively and accurately distinguish between the anterior and posterior channels based on the SSEP characteristics. In the context of functional brain mapping, relying solely on anatomical information, without the inclusion of a signal propagation model, could present difficulties in precisely determining whether a boundary channel predominantly observes sensory-motor or motor signals.

These new methods and results contribute to our understanding of cortical organization.

The findings from this study shed light on the patterns observed in SSEP traces acquired from uHD EEG and contribute to our understanding of the functional organization of the somatosensory cortex. Further, the results can have implications for clinical applications and future research in the field of sensory neuroscience. The uHD EEG acquisition system, combined with methodologies proven in intracranial ECoG recordings, shows promising results in terms of spatial mapping. The non-invasive approach allows high-resolution neurophysiological mapping of the human brain in a safe, simple environment.

Future research should combine the uHD EEG approach with source reconstruction techniques. This would allow high-resolution neurophysiological mapping through the acquisition of neural activation using non-invasive brain recordings.

## Methods

This paper evaluates an ultra-high-density EEG (uHD EEG) system using flexible printed circuit boards (PCBs) with gold-plated electrode points. With an inter-electrode distance of 8.6 mm and an electrode diameter of 5.9 mm, the density of the electrodes is higher than commercially available EEG systems on the market.

**Figure 3:**
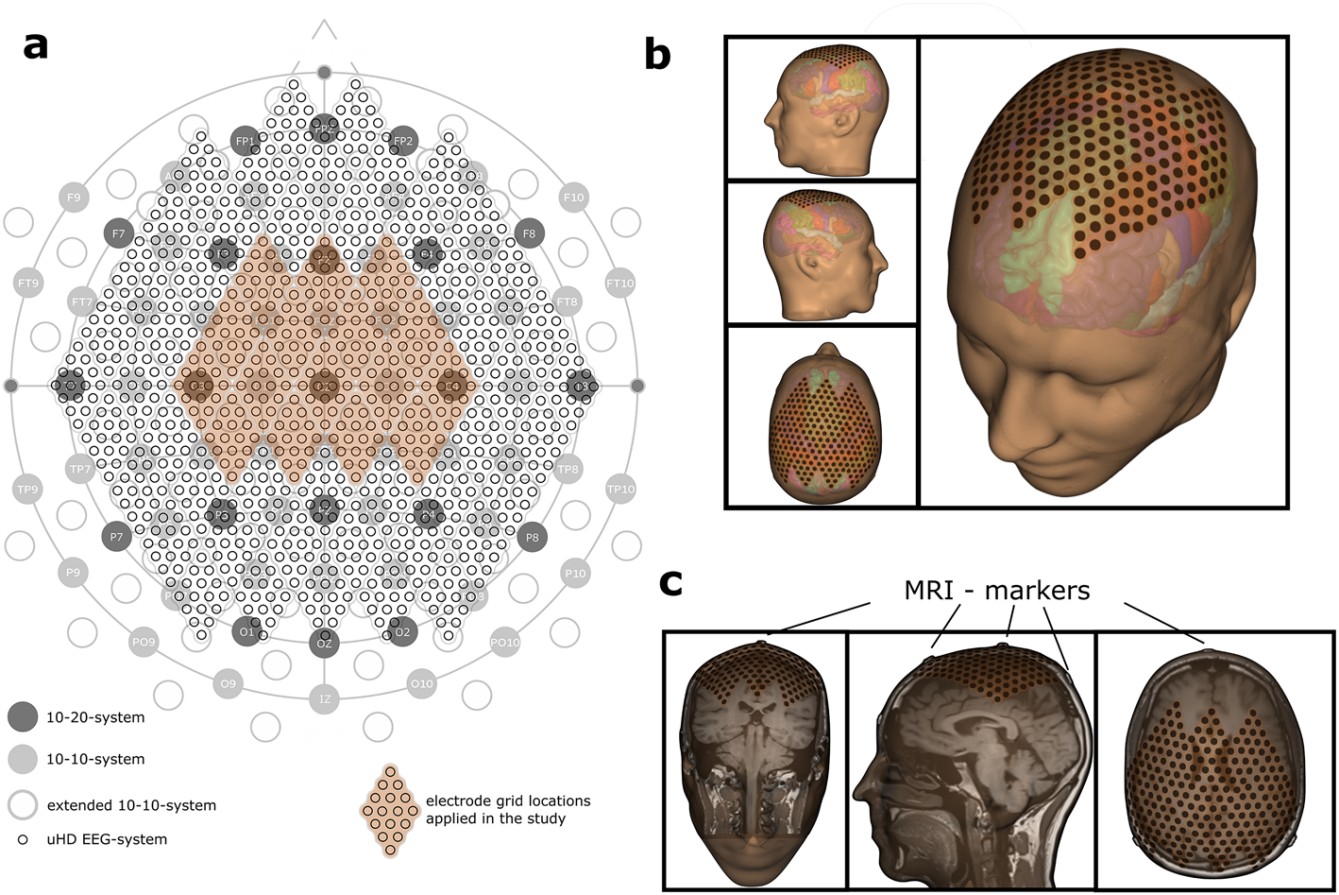
Electrode distribution of the uHD EEG system. (**a**) Comparison of the standard 10–20 EEG (dark gray circles with labels), the 10–10 system (light gray circles with labels), the extended 10–10 EEG (empty light gray circles), and the 1024 uHD EEG systems (small empty black circles). The orange area indicates the electrode positions used in this study (256 out of 1024 possible electrodes). (**b**) 3D head model of Subject S1 generated from a T1 weighted MRI scan. Electrode grids are marked in orange with the exact sensor locations indicated by the black dots. The head structure is slightly transparent, allowing the visualization of the left and right brain hemispheres, highlighted with the colors of the Desikan-Killiany Atlas. (**c**) Visible MRI markers in the coronal, sagittal, and axial planes. MRI markers are evident on the standard electrode positions CZ, PZ, and FZ.

Figure 3a depicts standardized electrode positioning systems. The 21 standard positions from the 10–20 system are dark grey. Figure 3a also includes two other systems: the 10–10 system (marked as filled light grey circles) and the extended 10–10 system (labeled as light grey circles). The uHD EEG system in this paper is illustrated by the small black circles in Fig. 3a and filled small black circles in Fig. 3b,c.

### Ultra-high-density EEG system

**Figure 4:**
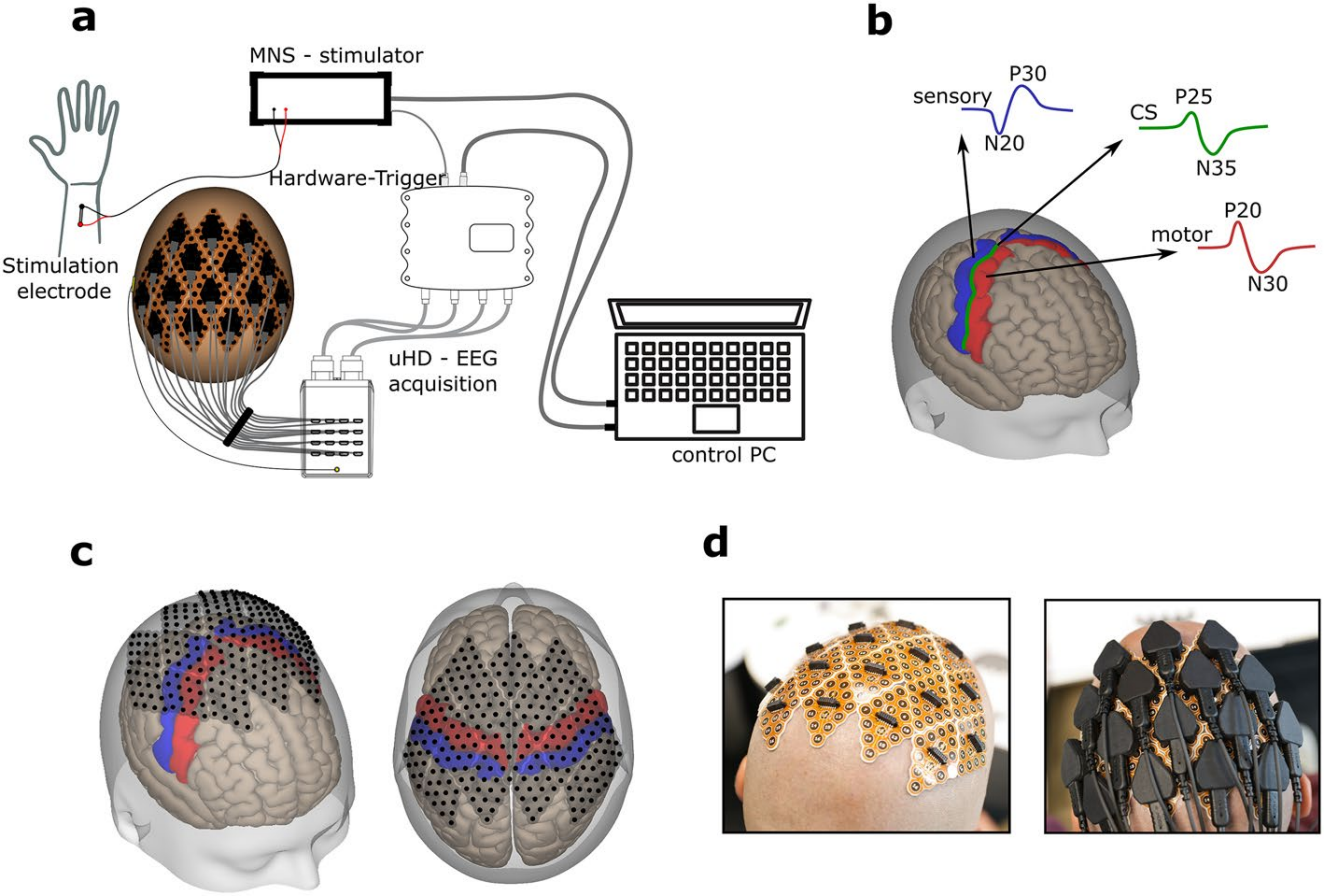
MNS using the 256-channel uHD EEG system. (**a**) Schematic representation of the system components as follows: uHD EEG acquisition system including the electrode grids, pre-amplifier, connector-box, and biosignal amplifier; MNS-stimulation device including bipolar stimulation electrodes; hardware trigger connector; control PC with acquisition software, (**b**) illustration of the MNI-brain with the areas of interest respectively marked: sensory-cortex (blue), motor-cortex (red), the estimated position of the central sulcus (green). Colored lines reflect typical neural responses after stimulation with the phase reversal from the motor (P20/N30) to the somatosensory area (N20/P30). (**c**) 256 electrode positions from the UHD-electrode grids marked on the scalp surface of the MNI brain. (**d**, left) Electrode grids on the prepared scalp without pre-amplifiers or cables. (**d**, right) Attached pre-amplifiers and cables for data transmission.

EEG was acquired using the uHD EEG system g.Pangolin (g.tec medical engineering GmbH, Schieldberg, Austria). This system can record from up to 1024 EEG channels distributed over the whole scalp. In this study, 256 channels were centrally placed and equally distributed over the left and right hemispheres of the sensorimotor cortex (see Figs. 3, 4a). The flexible electrode grids are attached to the skin using medical adhesives. The adhesive layer consists of moisture-resistant and insulating medical materials that prevent shortcuts and crosstalk. The recesses of the adhesive layer are filled with the conductive paste Elefix (Nihon Khoden, Tokyo, Japan) to ensure optimal skin contact and low impedance at the electrode-skin junction. The geometrical properties of the diamond-shaped electrode grids provide equal distribution of the channels over the curvature of the scalp and a constant inter-electrode distance. A pre-amplifier further improves signal quality and signal-to-noise ratio (SNR). The pre-amplifier is connected to the slim socket connector of the electrode grid (see Fig. 4d). The circuit board amplifies the signals with a fixed gain of 10. A connector box is used to interface the high-resolution electrode grids with the pre-amplifier and the biosignal amplifier. This interface box allows 256 channels to be recorded from the g.HIamp biosignal amplifier (g.tec medical engineering GmbH, Schieldberg, Austria). Data were acquired with a sampling frequency of 2400 Hz, which is necessary to capture the fast-rising edges expected from the MNS. EEG was recorded using a monopolar derivation with the Ground electrode placed on the mastoid using disposable Ag/AgCl electrodes Kendall™ H124SG (Cardinal Health, Dublin, Ireland).

### Electrode co-registration from MRI data

The topography plots were created using brain models generated from anatomical MRI scans of all five subjects. The brain and skull were reconstructed in FreeSurfer (Martinos Center for Biomedical Imaging, Cambridge, MA, USA) using T1-weighted MRI data^47^. For all subjects, the MRI scan was performed using a standard 10–20 cap equipped with MRI markers, and the resulting MRI artifacts were used for co-registration and electrode grid placement (see Fig. 3c). The electrode placement end co-registration procedure was performed using a custom montage creator software (g.tec Medical Engineering GmbH, Schiedlberg, Austria). The resulting montage contained 256 electrodes located centrally over the somatosensory and motor cortex (see Fig. 4c).

Using the head and brain model, we could accurately identify the motor and sensory areas that are highlighted by employing the Desikan–Killiany Atlas and are visually observable on the MRI cortex in these models. The central sulcus, marked in green (Fig. 4b), serves as the separation between the post- and pre-central gyrus. The ground truth channels were labeled accordingly through visual inspection.

### Subjects

Data were recorded from five healthy male adults, mean age 39 years (SD 7 years). No subject who participated in the measurements took any psychoactive medication or suffered from psychiatric or neurological disease. All participants had two healthy limbs and no sensory deficits. The subjects provided written informed consent, and the EEG studies were approved by the ethics commission from the medical faculty of the Johannes Kepler University (1256/2021). Each subject’s hair was shaved 1 day before measurement. The skin was cleaned with medical alcohol just before applying the electrode grids. Figure 4d depicts the electrode grid placement (total of 256 electrodes) on subject S1 and the application of the pre-amplifiers and cables for signal acquisition.

### Median nerve stimulation

The electrostimulation device g.Estim PRO (g.tec medical engineering GmbH, Schiedlberg, Austria) was utilized for MNS. A monophasic, alternating stimulation with a phase duration of 500 µs was applied to the middle of the wrist between the tendons to the flexor carpi radialis and palmaris longus. The stimulation electrode was a bipolar stainless-steel electrode with a fixed inter-electrode distance of 30 mm, an electrode diameter of 4.00 mm, and an exposed diameter of 2.30 mm, resulting in an exposed stimulation area of 4.16 mm^2^. The stimulation cathode was placed distally, and the anode was located more proximally.

The phase amplitude was set between 10 and 15 mA to a comfortable threshold for independently eliciting thumb movement in each subject. A pulse rate of 1.4 Hz, which results in a stimulus every 714 ms, was set for electrostimulation. The number of pulses was selected to be 300 to have sufficient data for averaging the SSEPs. Each subject experienced stimulation on both hands to provide results for electrodes located on both hemispheres. Hardware trigger impulses were led back from the stimulation device to the biosignal amplifier, which was converted to digital triggers to align the data for SSEP averaging.

Additionally, data were corrected for analog signal line delay due to the Analog Digital Converter (ADC) between the analog input and digital input lines. Due to the intrinsic filtering and down sampling of the ADC for analog channels, there is a delay between the analog biosignal input and digital trigger input lines. Therefore, with a sampling frequency of 2400 Hz, we corrected the data by six samples, as stated in the biosignal amplifier manual. The stimulation artifacts caused by MNS and the effects of pulse polarity were investigated and can be found in Supplementary Fig. S1.

### Signal-processing pipeline

**Figure 5:**
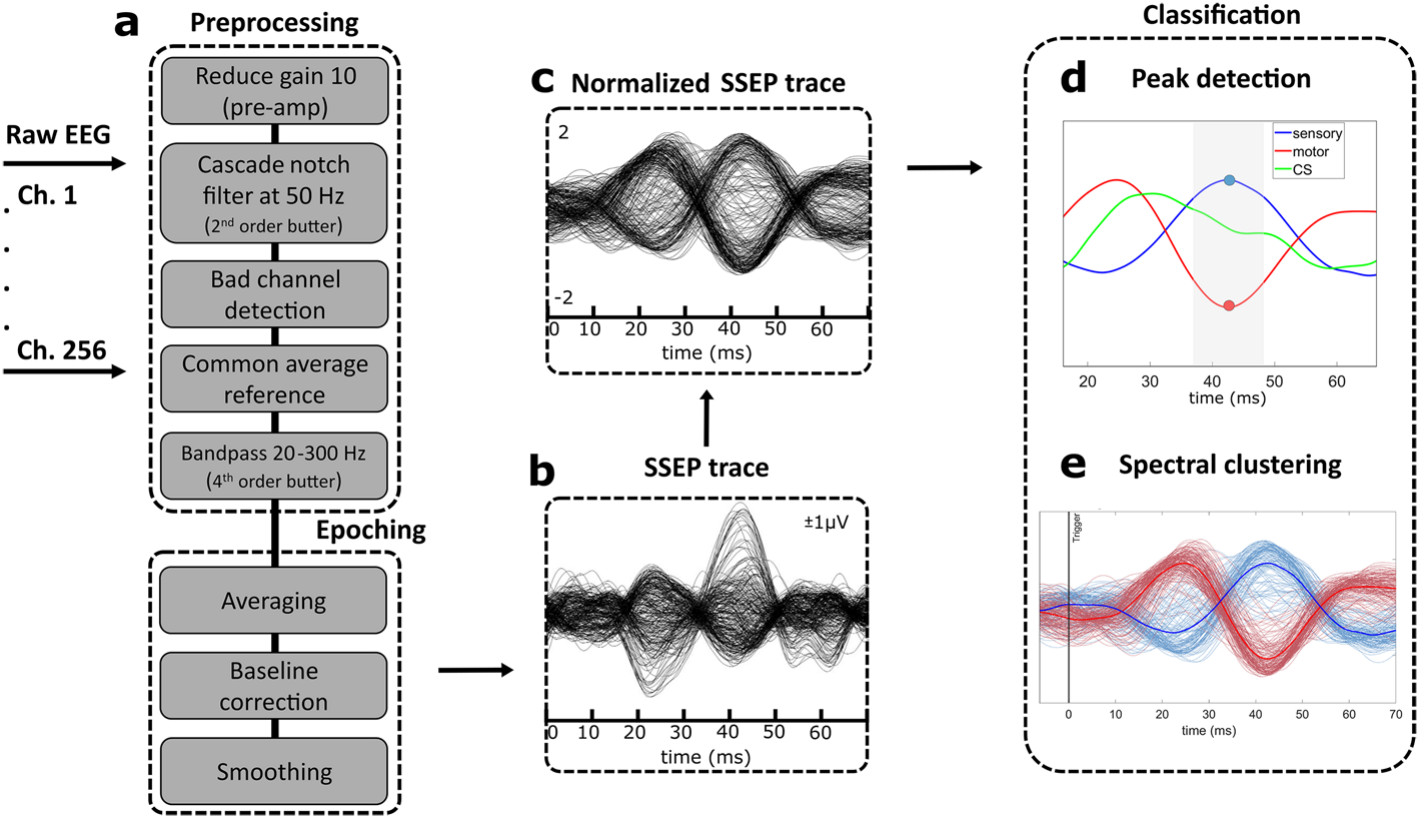
The EEG signal processing pipeline. 256-channel EEG data are recorded at a sampling frequency of 2400 Hz. (**a**) Pre-processing steps include cascaded notch filtering at 50 Hz (2nd order Butterworth), bandpass filtering from 20 to 300 Hz (4th order Butterworth), and Common Average Referencing (CAR) after excluding bad channels. After pre-processing, the data were epoched and averaged, with baseline correction applied. (**b**) The resulting SSEP traces were extracted, smoothed, and (**c**) normalized. (**d**) The peak information was used to plot heatmaps to locate the sensory-motor cortex and central sulcus. (**e**) Spectral clustering using k-means was performed to delineate the central sulcus. Blue represents sensory channels, red depicts motor channels, and green is the CS, with the channel average in bold.

The signal processing pipeline was implemented in MATLAB (The MathWorks, Inc., Natick, MA, USA) and is depicted in Fig. 5, including (a) pre-processing and (b) extracting the SSEP traces (c) normalizing SSEPs for (d) peak detection and (e) channel classification. As a general note, all filters were applied forwards and backward over the data to prevent phase shift using the MATLAB *filtfilt* function.

First, the gain factor of the pre-amplified EEG was removed. Second, data were filtered with a 50 Hz cascade Notch filter (2nd order Butterworth) to remove power line noise. Third, the data were common average referenced and filtered using a bandpass filter from 0.5 to 30 Hz (2nd order Butterworth) to detect bad channels. Fourth, the mean power was computed for each channel by subtracting their respective means, calculating the power by the squaring of each time sample, and calculating the mean power over time. Fifth, the mean channel powers were log-transformed to improve Gaussianity and z-transformed. Sixth, channels with a z-score greater than six were considered bad. Bad channels found using this procedure were removed from the notch-filtered EEG data, which were then common average referenced.

Referencing SSEP recordings is essential and extensively discussed in the literature. Mastoid, earlobe, FZ, or FPZ are conventionally recommended as reference sensor locations^33^. However, the average reference approach has also been effective^48,49^. Tsuchimoto et al. showed that common average reference (CAR) usage was superior to 7 other referencing methods when extracting sensorimotor activity^50^. The impedance mismatch can be significant from electrode arrays to the reference electrode. Hence, the representation of noise in both electrodes differs and thus is not entirely removed with conventional reference subtraction^49,51^. CAR was used in this study because the uHD EEG system does not provide additional electrode placement beside the grids in the areas of interest.

As found in the literature, different filter methods were tested for pre-processing the data^30,52^. A 20–300 Hz bandpass was selected based on the resulting SSEP traces and suggestions by the International Society of Interoperative Neurophysiology for the scalp (cortical and subcortical) SSEP recordings^3^. After processing described above, data were epoched into trials with a pre-trigger time of 100 ms and a post-trigger window of 300 ms, as these bins contain all of the information needed for SSEP analysis. 300 stimulation pulses were applied to the forearm, resulting in 300 trials used for averaging. The average of each channel was calculated and plotted, as seen in the SSEP trace from Fig. 5b. Plots in Fig. 5 represent SSEP traces acquired from the MNS to the right hand of Subject S1.

It is noteworthy that by visual inspection, the phase reversal is less conspicuous in electrodes positioned above the central sulcus compared to those in closer proximity to the motor and sensory cortex. However, regardless of the clearness of the trace, all SSEP traces were used for classification.

Independent component analysis (ICA)^53^ was also performed to detect the stimulation artifact to the abovementioned pipeline. Due to the lowpass filtering at 300 Hz, re-referencing using CAR, and alternating monopolar MNS, the artifacts were eliminated from the data used for further processing. Therefore, no components had to be removed from the data for further analysis (see Supplementary Fig. S1).

### Channel selection from left and right-hand MNS

The uHD electrode locations were placed centrally and partly on the left and right hemispheres (see Fig. 3a: around standard 10–20 positions CZ, C3, and C4). Data from both the right and the left-hand MNS had to be merged. Several studies have shown that the evoked potential measured from the contralateral hemisphere of the stimulated hand side is more reliable and consistent for estimating the activation source when performing Median nerve stimulation than the ipsilateral hand^32,54^. In contrast to the secondary somatosensory cortex S2, S1 appears to be selectively activated on the contralateral side of the stimulated hand. This observation supports the notion that S1 plays a crucial role in the processing of proprioceptive input^32^.

Therefore, we chose to select the electrode locations according to the hand side of the performed MNS, meaning that the dataset was merged by using the averaged SSEPs from channels located on the left hemisphere for right-hand MNS and using the averaged SSEPs from channels situated on the right hemisphere from the left hand MNS trials. The electrode split was performed along the longitudinal fissure. Electrode numbers were thereby selected as either left- or right-hemisphere sensor locations according to the co-registration procedure.

### Delineation using peak detection

Figure 5d shows an exemplary plot that distinguishes channels located in the sensory and motor areas. The plots represent the normalized and averaged SSEP traces of subject S1 with channel positions over the central sulcus (green) and the somatosensory (blue) and motor (red) cortices.

In the first step, channels were grouped according to the ground truth of being located anterior and posterior to the CS. From both averaged groups, a simple peak detection revealed the first and second peaks of the SSEP traces. The time point of the second peak was chosen for further analysis of all channels independently. Data for each channel were then selected from the time window where ${X}_{\text{frame}}^{(i)}$ is the subset of ${X}^{\left(i\right)}\left(t\right)$ for each channel i that is situated within the time frame of the selected start (${t}_{\text{start}}=t-5\text{ ms})$ and end time ${(t}_{\text{end }}=t+5\text{ ms}).$

1 $${{\text{X}}}_{\text{frame}}^{({\text{i}})}=\left\{{{\text{X}}}^{\left({\text{i}}\right)}({\text{t}})|{{\text{t}}}_{\text{start}}\le {\text{t}}\le {{\text{t}}}_{\text{end}}\right\}.$$

Using the subset in the range of indices as described in Eq. (1), we can distribute the data around zero by subtracting the mean from each value. Finally, data were normalized by dividing it by the maximum absolute difference, ensuring the values fall within a range of − 1 and 1 (see Eq. 2).

2 $${\text{y}}\left({\text{i}}\right)=\frac{{{\text{X}}}_{\text{frame}}^{({\text{i}})}-\text{mean }\left({{\text{X}}}_{\text{frame}}^{({\text{i}})}\right)}{{\text{max}}\left|{{\text{X}}}_{\text{frame}}^{({\text{i}})}-{\text{mean}}\left({{\text{X}}}_{\text{frame}}^{({\text{i}})}\right)\right|}.$$

To classify channels, the MATLAB *findpeaks* function was applied to detect positive and negative peaks and peak amplitudes from ${\text{y}}\left(i\right)$. The resulting vector was given as input for creating topographies for delineating the central sulcus. Additionally, the classification accuracy was calculated as shown in Eq. (4). Subsequently, all channels with positive values in the vector y(i) were labeled as Pst. (sensory), while those with negative values were labeled as Ant. (motor) channels and compared to the ground truth.

### Classification using spectral clustering

Figure 5e presents a schematic visualization of the SSEP traces classified using the spectral clustering methodology adopted from Asman et al.^30^. All 256 normalized SSEP traces from S1 are depicted (bad channels excluded). Channels located posterior to the CS are marked in blue, with the mean of all channels marked in bold blue. Channels anterior to the CS are drawn in red, with their mean in bold red. To preserve the waveform morphology, we normalized the SSEP traces and applied spectral clustering to group the data in an unsupervised manner. Spectral clustering is a machine-learning technique that does not make any assumptions about the shapes of the clusters^55,56^. As described by Asman et al.^30^, a Gaussian similarity function was used, Eq. (3), to create the adjacency matrix ${W}_{ij}$:

3 $${W}_{ij}={e}^{\frac{{-\left|{x}_{i}-{x}_{j}\right|}^{2}}{{2\sigma }^{2}}}.$$

The data segmentation was inferred using spectral graph theory, which involved applying k-means to the second smallest eigenvector of the normalized Laplacian matrix. The graph Laplacian was normalized using the random walk method^30,57^. The adjacency matrix represented the connectivity network between different EEG channels. The matrix entries were calculated using the Euclidean distance between the normalized SSEP traces, ${x}_{i}$ and ${x}_{j}$, of channels $i$ and $j$. To determine the appropriate value of the standard deviation, $\sigma$, the algorithm was run multiple times with different values of $\sigma$ as suggested by Ng et al.^58^. The value of $\sigma$ = 4 was selected because it resulted in the fewest distorted clusters of the normalized trace across all subjects.

### Classification performance

We calculated the number of correctly classified anterior and posterior channels based on the ground truth. We computed the confusion matrix for true and predicted classes. True positives (TP), true negatives (TN), false positives (FP), and false negatives (FN) were then used to determine the accuracy as defined in Eq. (4):

4 $${\text{Accuracy}}= \frac{({\text{TP}}+{\text{TN}})}{({\text{TP}}+{\text{FP}}+{\text{TN}}+{\text{FN}})}.$$

### Ethics declarations

All experiments to collect the datasets were performed in accordance with the Declaration of Helsinki, and the experimental procedures were approved by the Ethics Committee of the Faculty of Medicine of the Johannes Kepler University, Linz (number: 1256/2021). Written informed consent to participate in the present study was obtained from every participant.

### Supplementary Information

Supplementary Information.

## Data Availability

A sample dataset from the median nerve stimulation using the uHD EEG system is available on https://osf.io/srzam/files/osfstorage/646239a47b916a439b2997d5. Complete datasets collected from the central sulcus mapping experiments are available upon reasonable request. Please contact schreiner@gtec.at for any inquiries or requests related to the data from this study.
