# Supplementary material for: Mapping of the central sulcus using non-invasive ultra-high-density brain recordings
Source: Sci Rep. 2024 Mar 19;14:6527. doi: 10.1038/s41598-024-57167-y (PMC10948849; doi:10.1038/s41598-024-57167-y)
Supplement: Supplementary file 1 — Supplementary Information. [file 41598_2024_57167_MOESM1_ESM.docx]

**Supplementary Information**

**Mapping of the central sulcus using non-invasive ultra-high-density brain recordings**

**Supplementary Methods: Independent Component Analysis (ICA) for detection of the stimulation artifact**

Median nerve stimulation (MNS) simultaneously acquiring EEG causes visible stimulation artifacts in the peripheral electrophysiological data. These stimulation artifacts are of non-physiological origin and need to be assessed^1^. Different approaches can be used to remove these artifacts, such as cutting and interpolation^2^ or applying ICA for extracting and removing the artifact components^3^.

In the present study, the stimulation artifact was detected using the FastICA algorithm by Hyvarinen et al.^3^. The raw EEG data were high-pass filtered at 1 Hz using a 2^nd^ order Butterworth filter, and the filter coefficients were applied using *filtfilt* in MATLAB and then provided as input to the FastICA. Additionally, the number of independent components to be estimated was limited to 50. This was done to keep the computational load small, as trying to estimate 256 independent components is not necessary for this analysis.

Fig. S1a shows the stimulation artifact component for the first 25 stimulations, estimated by the FastICA algorithm for subject S2 during their left MNS. Subject S2 was chosen, as their data had strong stimulation artifacts. Note that the polarity of these stimulation artifacts is alternating, which is caused by the alternating MNS polarity.

Fig. S1b shows the power spectral density (PSD) of the stimulation artifact component for all 300 stimulations, estimated using Welch’s method. Specifically, windows of 10 seconds with a step size of 0.1 seconds were used, and a Hann window was applied to reduce spectral leakage. Next to the 50 Hz power line noise and its harmonics, numerous small peaks, which are integer multiples of 1.4 Hz, can be observed (i.e., harmonics of 1.4 Hz). These peaks are caused by the stimulation at a pulse rate of 1.4 Hz.

This phenomenon is further illustrated in Fig. S1c, which shows the epoched independent component for all 300 trials, with a 5 ms pre- and post-trigger time window. Negative deflections of the stimulation artifacts reflect positive MNS pulses and vice versa. Averaging of all 300 trials practically eliminates the stimulation artifact as shown by the black line in Fig. S1c. In sum, the stimulation artifact can be detected using the FastICA algorithm and is present in single trials. However, alternating the polarity of the stimulation and averaging over a large number of trials practically eliminates the stimulation artifact. Additionally, filters with a low-pass characteristic, such as the band-pass filter used for extracting the EPs attenuate the stimulation artifact.


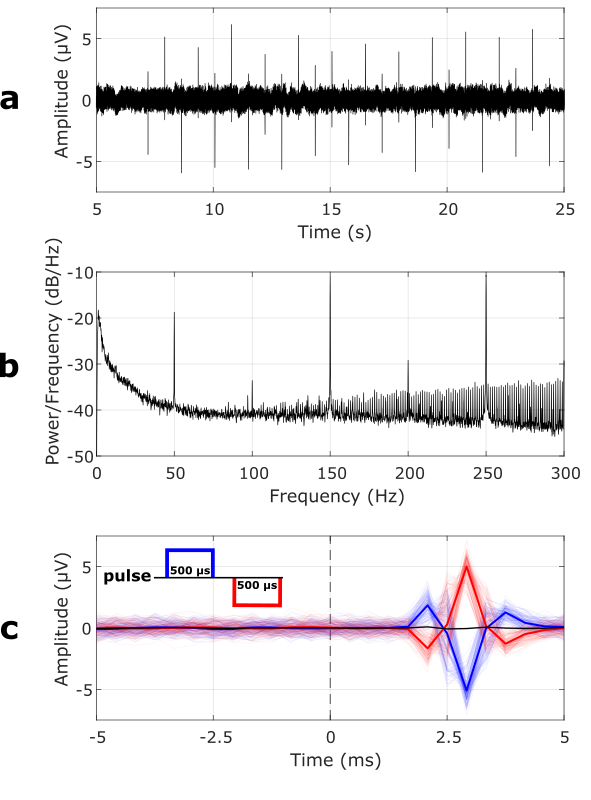


**Supplementary Figure S1: Stimulation artifact detection using ICA. (a)** The stimulation artifact component contains artifacts with a 1.4 Hz pulse rate. **(b)** The power spectral density provides additional proof of artifacts by assessing the numerous small peaks integer multiples of 1.4 Hz (i.e., harmonics of 1.4 Hz). Also visible is the 50 Hz power line noise and its harmonics. **(c)** The alternating polarity of the stimulation with 150 trials of positive (blue) and 150 trials of negative (red) MNS stimulation pulse. The delay of the stimulation artifact represents the analog signal line between analog and digital input lines.

A delay of ~2.5 ms of the stimulus impulse can be seen in Fig. S1c. The delay is a result of differences between analog input and digital input lines. Data were led back from the stimulation device to the biosignal amplifier, which was converted to digital triggers to align the data for SSEP averaging. Due to the intrinsic filtering and downsampling of the Analog Digital Converter (ADC) for analog channels, there is a delay between the analog biosignal input and digital trigger input lines. With a sampling frequency of 2400 Hz we corrected the data by 6 samples (2.5ms) as stated in the biosignal amplifier manual.

**References**

1. Hoffmann, U., Cho, W., Ramos-Murguialday, A. & Keller, T. Detection and removal of stimulation artifacts in electroencephalogram recordings. in 2011 Annual International Conference of the IEEE Engineering in Medicine and Biology Society 7159–7162 (2011). doi:10.1109/IEMBS.2011.6091809.

2. Stephani, T., Nierula, B., Villringer, A., Eippert, F. & Nikulin, V. V. Cortical response variability is driven by local excitability changes with somatotopic organization. 2022.04.26.489557 Preprint at https://doi.org/10.1101/2022.04.26.489557 (2022).

3. Hyvarinen, A. Fast and robust fixed-point algorithms for independent component analysis. IEEE Trans. Neural Netw. 10, 626–634 (1999).
